# Structural insights into WcbI, a novel polysaccharide-biosynthesis enzyme

**DOI:** 10.1107/S205225251302695X

**Published:** 2013-10-18

**Authors:** Mirella Vivoli, Emily Ayres, Edward Beaumont, Michail N. Isupov, Nicholas J. Harmer

**Affiliations:** a College of Life and Environmental Sciences, University of Exeter, Geoffrey Pope Building, Stocker Road, Exeter EX4 4QD, England

**Keywords:** capsular polysaccharides, acetyl-CoA, acetyltransferases, *Burkholderia pseudomallei*, melioidosis

## Abstract

Capsular polysaccharides (CPSs) are protective structures on the surfaces of many Gram-negative bacteria. The principal CPS of the human pathogen and Tier 1 select agent *Burkholderia pseudomallei* consists of a linear repeat of –­3)-­2-*O*-acetyl-6-deoxy-β-d-*manno*-heptopyranose-(1–. This CPS is critical to the virulence of this emerging pathogen and represents a key target for the development of novel therapeutics. *wcbI* is one of several genes in the CPS biosynthetic cluster whose deletion leads to significant attenuation of the pathogen; unlike most others, it has no homologues of known function and no detectable sequence similarity to any protein with an extant structure. Here, the crystal structure of WcbI bound to its proposed product, coenzyme A, is reported at 1.38 Å resolution, solved using the halide-soak method with multiple anomalous dispersion. This structure reveals that WcbI incorporates a previously described 100-amino-acid subdomain into a novel, principally helical fold (310 amino acids). This fold adopts a cradle-like structure, with a deep binding pocket for CoA in the loop-rich cradle. Structural analysis and biophysical assays suggest that WcbI functions as an acetyltransferase enzyme, whilst biochemical tests suggest that another functional module might be required to assist its activity in forming the mature *B. pseudomallei* capsule.

## Abstract 2

The structure of WcbI, a protein essential to the virulence of *B. pseudomallei*, reveals that this protein has a novel fold. WcbI binds strongly to coenzyme A, but appears to require an unidentified partner protein for its putative acetyltransferase function.

## 1. Introduction   

The human pathogen *Burkholderia pseudomallei* is a Gram-negative soil-dwelling bacterium that causes a severe febrile disease, melioidosis. The organism is endemic throughout the tropics (Wiersinga *et al.*, 2012 ▶) and is the most common form of community-acquired infection in parts of Thailand and Northern Australia. It has been reported on every continent except Antarctica (Stone, 2007 ▶). *B. pseudomallei* infects humans, a range of animals and some plants. Disease states in humans include acute and chronic forms, ranging from acute pneumonia or septicaemia to chronic abscesses (Lazar Adler *et al.*, 2009 ▶); without the use of appropriate antibiotics, the septicaemic form of melioidosis has a mortality rate that exceeds 90% (Warner *et al.*, 2007 ▶). Many countries in which *B. pseudomallei* is endemic are located in less developed parts of the world, where bacteria cause disease in large numbers of people. It is believed that between 20 and 50% of all patients diagnosed with severe forms of melioidosis will die, depending on their access to appropriate medicines and healthcare, which is often not sufficient in less developed countries. Even with satisfactory healthcare, relapse of the disease is a common problem, with many patients requiring treatment for the rest of their lives (Lazar Adler *et al.*, 2009 ▶). Because of its high mortality rate, *B. pseudomallei* is considered as a potential bioterror threat and is currently classed by the US Centers for Disease Control (CDC) as a Tier 1 select agent (Department of Health and Human Services, 2012 ▶). *B. pseudomallei* is naturally resistant to many antibiotics, and has no currently available vaccine. An effective vaccine to both protect populations exposed to infection and to alleviate the risk of bioterrorism is thus urgent.

The principal capsular polysaccharide (CPS-I) of *B. pseudomallei* is one of the best verified virulence factors of this organism (Lazar Adler *et al.*, 2009 ▶; Wiersinga *et al.*, 2006 ▶) and is considered to be a strong candidate for vaccine development. Ablation of CPS-I abolishes *B. pseudomallei* virulence in animal models (Reckseidler *et al.*, 2001 ▶; Atkins, Prior, Mack, Russell, Nelson, Prior *et al.*, 2002 ▶), and CPS provides partial protection when used to vaccinate mice against melioidosis (Nelson *et al.*, 2004 ▶). Most strains of the non-pathogenic close relative organism *B. thailandensis* do not have the genes required for CPS biosynthesis (Reckseidler *et al.*, 2001 ▶), and live vaccines consisting of mutants lacking a polysaccharide capsule have been shown to provide only partial protection against melioidosis (Atkins, Prior, Mack, Russell, Nelson, Oyston *et al.*, 2002 ▶; Sarkar-Tyson *et al.*, 2009 ▶; Stevens *et al.*, 2004 ▶; Breitbach *et al.*, 2008 ▶).

**Figure 1:**
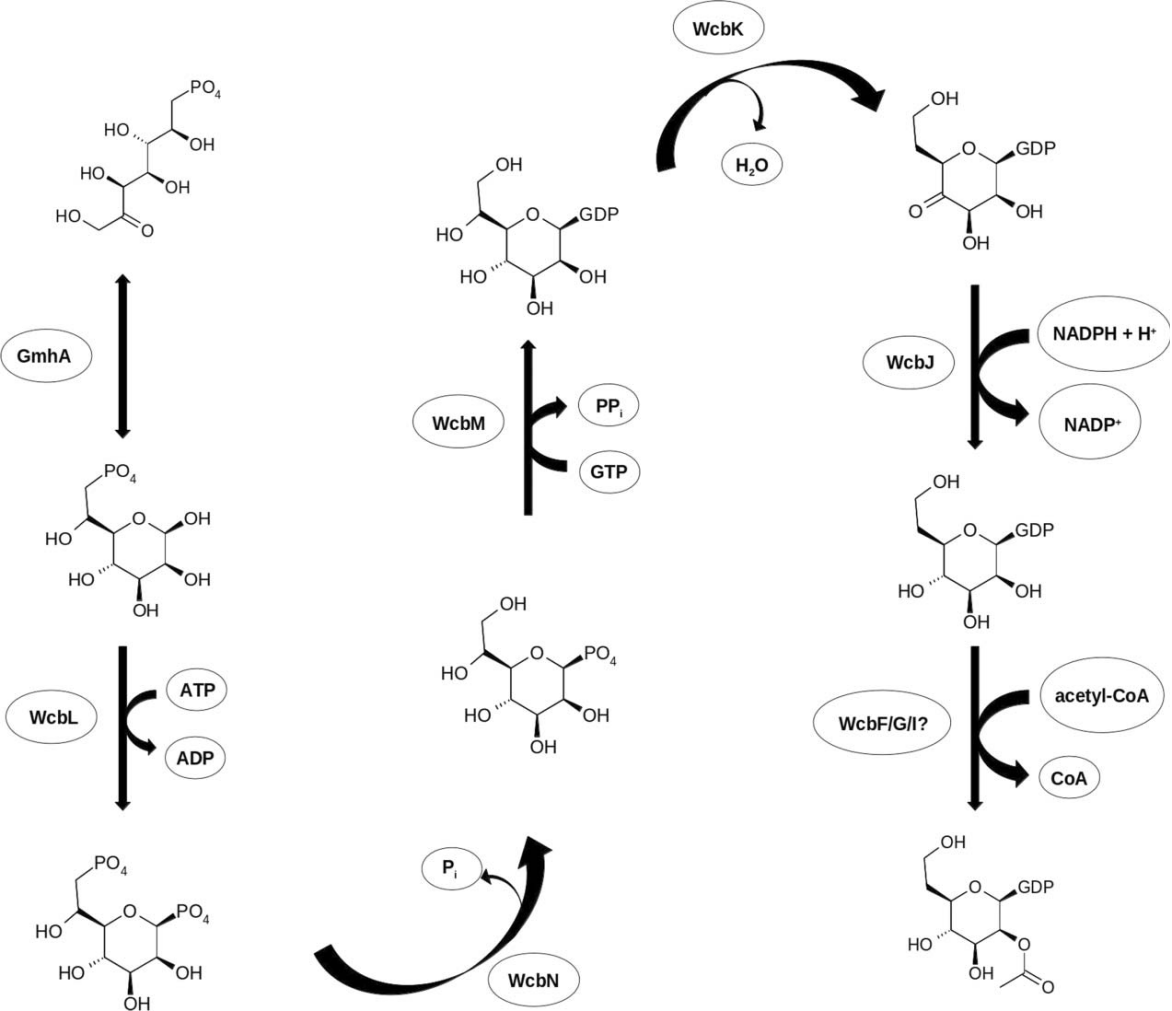
Proposed pathway for the biosynthesis of 2-*O*-acetyl-6-deoxy-β-d-*manno*-heptopyranose from sedoheptulose 7-phosphate in *B. pseudomallei* (after Harmer, 2010 ▶).

CPS-I of *B. pseudomallei* and the related Tier 1 select agent *B. mallei* consists of a linear polymer of –3)-2-*O*-acetyl-6-­deoxy-β-d-*manno*-heptopyranose-(1– (2Ac-dDHep; Reckseidler *et al.*, 2001 ▶; Heiss *et al.*, 2012 ▶). This polysaccharide is biosynthesized by a cluster of 24 genes found on *B. pseudomallei* chromosome 1 (Reckseidler *et al.*, 2001 ▶; DeShazer *et al.*, 2001 ▶; Holden *et al.*, 2004 ▶). The function of the majority of these has been proposed from bioinformatics and functional studies (Cuccui *et al.*, 2012 ▶). However, three genes (*wcbF*, *wcbG* and *wcbI*) do not have proposed functions, and the protein responsible for 2-*O*-acetylation of the capsule sugars has not been identified (Fig. 1 ▶). *wcbI* has been shown to be required for virulence (DeShazer *et al.*, 2001 ▶; Cuccui *et al.*, 2007 ▶). In addition, the location of the gene in this cluster, and the diminished capsule manufacture in its absence, suggest that this gene is necessary for efficient CPS-I production. *BLAST* searches of the sequence do not reveal any homologues with predicted functions, and a series of fold-recognition servers give only equivocal matches to known structures.

To determine the biochemical function of WcbI, we have therefore solved its crystal structure to 1.38 Å resolution. The structure revealed that WcbI adopts a predominantly helical fold: the N-terminal 100 amino acids form a ligand-binding domain fold, with the remaining 210 amino acids adopting an entirely novel fold. The protein binds tightly to coenzyme A and its derivative acetyl-CoA, with an apparent *k*_d_ of 58 µ*M* by differential scanning fluorimetry. Our extensive biochemical assays failed to show acetyltransferase activity for WcbI when incubated with any available substrate. Based on the structure and on biochemical and biophysical data, we propose that WcbI functions as an acetyltransferase and requires another functional module to carry out this function.

## 2. Materials and methods   

### 2.1. Protein cloning, expression and purification   

Full-length *wcbI* from *B. pseudo­mallei* strain K96423 (the genomic DNA was a gift from R. Titball, University of Exeter) was cloned into pNIC28-Bsa4 (Savitsky *et al.*, 2010 ▶; a gift from O. Gileadi, SGC Oxford). Constructs were transformed into *Escherichia coli* Rosetta (DE3) cells (Merck) and grown in ZYM-5052 medium (Studier, 2005 ▶) supplemented with 100 µg ml^−1^ kanamycin and 20 µg ml^−1^ chloramphenicol. Cells were grown at 37°C until the OD_600_ reached 0.5 and then at 20°C for 16 h. The harvested cells were resuspended in 20 m*M* Tris–HCl pH 8.0, 0.5 *M* NaCl (buffer *A*) and lysed using a Soniprep 150 sonicator (MSE). The clarified lysate was purified using a nickel–agarose column (Bioline) or a HisTrap Crude FF column (GE Healthcare). Briefly, the loaded protein was washed with buffer *A* supplemented with 25 m*M* imidazole–HCl pH 8.0 and eluted with buffer *A* supplemented with 250 m*M* imidazole–HCl pH 8.0. WcbI was then loaded onto a Superdex 200 16/60 HR column (GE Healthcare) and eluted isocratically with 10 m*M* HEPES pH 7.0, 0.5 *M* NaCl. For preparation of tag-free WcbI, purified protein was incubated at 4°C for 48 h with 1:100 TEV protease (S219V mutant; Kapust *et al.*, 2001 ▶; purified using methods available at http://mcl1.ncifcrf.gov/waugh_tech/protocols/pur_histev.pdf). The sample was then extensively loaded over nickel–agarose to remove residual undigested WcbI and TEV.

### 2.2. Crystallization and structure determination   

**Figure 2:**
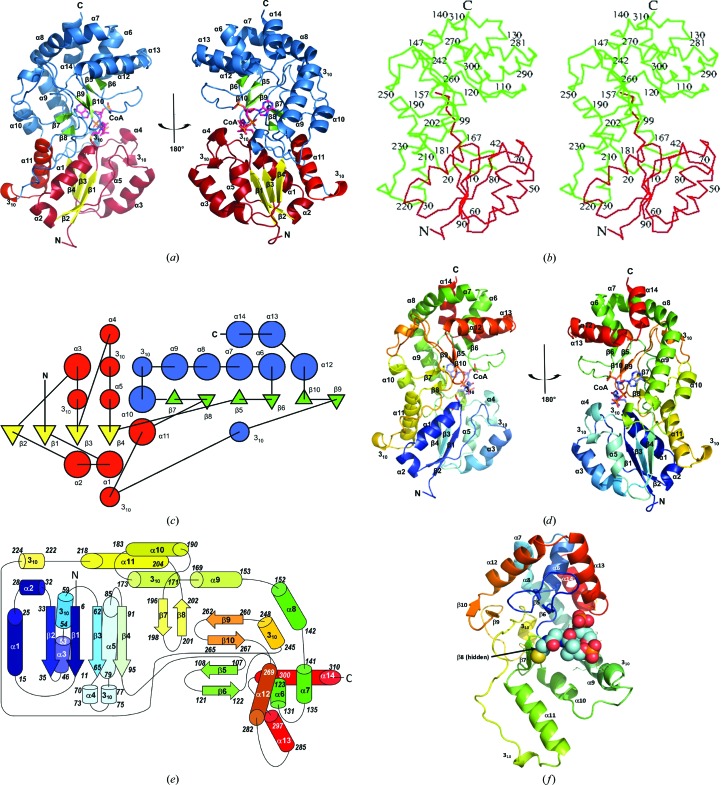
The structure of WcbI reveals a novel fold. (*a*) Cartoon representation of the crystal structure of WcbI in two orientations. Termini and secondary-structure elements are labelled. For the N-terminal subdomain α-helices and loops are shown in red and β-sheets in yellow; for the C-terminal subdomain α-helices and loops are shown in blue and β-sheets in green. (*b*) Stereoview of the C^α^ backbone in the same orientation as in the left-hand image of (*a*). Every tenth residue is numbered. (*c*) Topological diagram of the polypeptide fold with α-helices indicated by circles and β-strands by triangles coloured as in (*a*). (*d*) Cartoon representation of WcbI in the orientations in (*a*) coloured as a rainbow with the N-terminus in blue and the C-terminus in red. (*e*) Schematic diagram of the fold of WcbI. α-Helices and 3_10_-helices are shown as cylinders and β-sheets are shown as arrows. Secondary-structure elements are coloured as in (*d*). The start and end residue of each element is indicated. (*f*) Cartoon representation of the novel fold C-terminal subdomain coloured as a rainbow with the N-terminus in blue and the C-terminus in red. The substrate coenzyme A is shown as spheres. Carbon, cyan; oxygen, red; nitrogen, blue; sulfur, yellow; phosphorus, orange. Interacting residues from the novel subdomain are shown as lines. The secondary-structure assignments in all panels were made according to *TOPS* (Michalopoulos *et al.*, 2003 ▶). (*a*), (*b*), (*d*) and (*f*) were prepared with the *PyMOL* Molecular Graphics System (Schrödinger) and (*c*) was prepared with TOPS.

**Figure 3:**
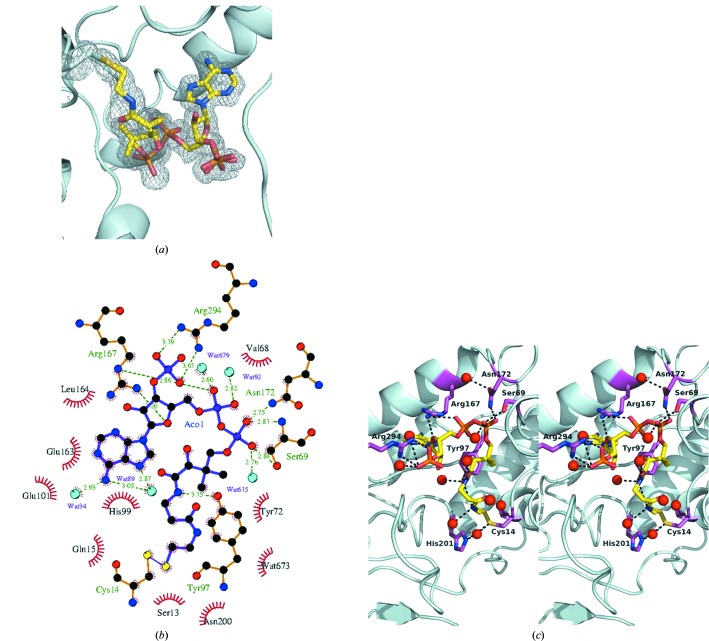
WcbI binds to coenzyme A. (*a*) Electron-density map showing the binding of coenzyme A (CoA) to WcbI. WcbI is shown as a cyan cartoon, with CoA as sticks. For clarity, water molecules are not shown. An *F*_o_ − *F*_c_ OMIT map is shown contoured at 3σ. The bond from CoA to Cys14 is shown. (*b*) Schematic drawing of the WcbI–CoA interactions. (*c*) Key WcbI–CoA interactions. WcbI is shown as a cyan cartoon, with interacting side chains as purple sticks. CoA is shown as sticks. Hydrogen-bond distances are shown. Nitrogen, blue; oxygen, red, phosphorus, orange; CoA carbon, yellow; WcbI carbon, purple. (*a*) and (*c*) were prepared with the *PyMOL* Molecular Graphics System (Schrödinger) and (*b*) was prepared with *LigPlot* ^+^ (Laskowski & Swindells, 2011 ▶).

All crystals were grown using the microbatch method and were prepared using an Oryx 6 crystallization robot (Douglas Instruments). Crystals grew in 2.5 mg ml^−1^ WcbI, 10%(*w*/*v*) PEG 8000, 75 m*M* LiCl, 75 m*M* MgCl_2_, 0.05 *M* HEPES pH 7.0–8.0 at 4°C. Prior to flash-cooling, crystals were soaked in cryoprotectant solutions consisting of 22%(*v*/*v*) PEG 400, 8%(*v*/*v*) PEG 8000, 1 *M* NaBr, 0.05 *M* HEPES pH 7.0, 7.5 and 8.0 for 5 min. Co-crystallization with acetyl-CoA was achieved by growing crystals in the same conditions, with WcbI at 2 mg ml^−1^, supplemented with 200 µ*M* acetyl-CoA. These crystals were cryopreserved in the above solution without sodium bromide. The WcbI structure was solved using the multiple anomalous dispersion (MAD) method following the short (<5 min) cryosoaking of a crystal with bromide ions (Dauter *et al.*, 2000 ▶). Diffraction data were collected from a bromide-soaked crystal on beamline I02 at the Diamond Light Source synchrotron at four wavelengths (Table 1 ▶) using an ADSC detector. Data were processed with *XDS* (Kabsch, 2010 ▶) and scaled together using *SCALA* (Evans, 2011 ▶) and the *xia*2 (Winter, 2010 ▶) pipeline. Most of the further data and model manipulations were carried out using programs from the *CCP*4 suite (Winn *et al.*, 2011 ▶). Anomalous scatterers were located and a polyalanine model of the protein was built using the *SHELXC*/*D*/*E* pipeline (Sheldrick, 2010 ▶) implemented in *HKL*2*MAP* (Pape & Schneider, 2004 ▶). A full atomic model was built using the *ARP*/*wARP*/*REFMAC*5 pipeline (Langer *et al.*, 2008 ▶; Murshudov *et al.*, 2011 ▶). High-resolution data were collected from the acetyl-CoA-soaked crystal on beamline I24 at Diamond Light Source using a PILATUS detector. The final refinement of both models was conducted with *PHENIX* (Adams *et al.*, 2010 ▶). The model was manually rebuilt in *Coot* (Emsley *et al.*, 2010 ▶) and the final model was validated using *PHENIX* and *MolProbity* (Chen *et al.*, 2010 ▶). The statistics of the refinement and the stereochemistry of the final models are summarized in Table 1 ▶. The coordinates and structure factors were deposited in the Protein Data Bank under accession codes 4bqn for WcbI covalently bound to coenzyme A and 4bqo for the native structure. Figs. 2(*a*), 2(*d*), 2(*f*), 3(*a*), 3(*c*) and Supporting Figs. S2(*a*), S2(*c*) and S3(*a*) were prepared with the *PyMOL* Molecular Graphics System (Schrödinger), and Fig. 3(*b*) and Supporting Figs. S2(*b*) and S3(*b*) were prepared with *LigPlot* ^+^ (Laskowski & Swindells, 2011 ▶). The stereoview of the WcbI C^α^ backbone (Fig. 2*b*) was prepared using the program *BobScript* (Kraulis, 1991 ▶; Esnouf, 1997 ▶) and Fig. 2(*c*) was prepared with *TOPS* (Michalopoulos *et al.*, 2003 ▶).

### 2.3. Differential scanning fluorimetry (DSF)   

DSF samples were prepared at the following concentrations in a final volume of 20 µl: 0.1 mg ml^−1^ protein, 10 m*M* HEPES pH 7.0, 150 m*M* NaCl, 8× SYPRO Orange dye (Invitrogen; Niesen *et al.*, 2007 ▶). Potential ligands were added to final concentrations of between 0.1 µ*M* and 1 m*M*. All samples were prepared in triplicate. Fluorescence was measured using a StepOne quantitative PCR machine (Applied Biosystems) while heating the samples in a gradient from 25 to 99°C over 40 min. Measurements were taken every 0.37°C. The DSF curves showed a mixture of two populations of protein. Rather than calculating the midpoint temperature of the unfolding transition (*T*_m_), the curves were fitted to determine the proportions of protein in the bound and unbound states. A full description of this approach will be published shortly (Vivoli & Harmer, in preparation). Briefly, the differential of each DSF curve was calculated using the *Protein Thermal Shift* software package (Applied Biosystems), and each curve was compared with the unbound sample and a sample at the highest ligand concentration to determine the proportion in a bound state using *SPSS* v.20. The dissociation constant *k*_d_ was calculated using
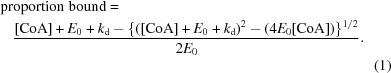

*E*_0_ was treated as a constant, and the value of *k*_d_ was determined using *SPSS* v.20. Following the calculation of *k*_d_, a standard curve was modelled using this equation. The likely proportion bound in the highest ligand concentration sample was calculated and a differential curve at 100% ligand concentration was modelled. The determination process was then repeated until the model converged. 29 concentrations of CoA were used in at least triplicate, and the results reported are representative of results obtained on at least three days.

### 2.4. Acetyltransferase kinetic assays   

#### 2.4.1. Assay of coenzyme A production by coupling to 5′-­dithio-bis(2-nitrobenzoic acid)   

The activity assay was based on the hydrolysis of acetyl-CoA in the presence of the substrates guanosine diphosphate mannose (GDP-mannose) or mannotetraose. 5′-Dithio-bis(2-nitrobenzoic acid) (DTNB) was used as a colorimetric developing agent for the detection of CoA (Shaw, 1977 ▶). Acetyl-CoA (0–1 m*M*), GDP-mannose (4 m*M*) or mannotetraose (2 m*M*) and 0.5 µ*M* purified recombinant WcbI were mixed and pre-incubated at room temperature for 5 min in a 96-well plate in a final volume of 50 µl containing 100 m*M* Tris–HCl buffer pH 8.0. The reaction was stopped with 25 µl guanidine hydrochloride solution (6.4 *M* guanidine–HCl, 0.1 *M* Tris–HCl pH 8.0) and incubated for 3 min, gently shaking. Finally, 25 µl 10 m*M* DTNB was added to the stopped reaction and the absorbance at 412 nm (peak of absorbance of 2-nitro-5-thiobenzoic acid; TNB) was measured. Rates were calculated using an extinction coefficient for TNB at 412 nm of 13 600 *M* ^−1^ cm^−1^. Reactions in which GDP-mannose, mannotetraose, acetyl-CoA or recombinant WcbI were omitted served as controls. The same assay was performed using 20 U chloramphenicol acetyltransferase (Sigma) and 2 m*M* chloramphenicol for a positive control.

#### 2.4.2. Assay of coenzyme A production using fluorescence   

The assay for the determination of acetyltransferase activity was carried out using the acetyltransferase activity kit (Enzo Life Sciences) as per the manufacturer’s instructions. Briefly, in a 96-well plate, 0–3 m*M* donor substrate (GDP-mannose) was diluted in 1× reaction mixture. An enzyme mixture consisting of 25 µl 1× transferase assay buffer and 25 µl 0.5 µ*M* WcbI was added to this. The plate was covered with a foil sealer and incubated for 5 min with constant shaking in an orbital shaker at room temperature. 50 µl positive control (supplied in the kit) was added to the appropriate wells. 50 µl ice-cold isopropyl alcohol was then added to each well. Finally, 100 µl of the 1× detection solution (supplied in the kit) was added to each well. The plate was covered with a foil sealer and further incubated for 10 min at room temperature without shaking. The plate was then read at 380 nm excitation and 520 nm emission wavelengths. The test was performed in duplicate. The signal-to-noise ratio was calculated from the relative fluorescence unit of a blank with buffer alone.

#### 2.4.3. Assay of coenzyme A production using a coupled reaction with pyruvate dehydrogenase   

Acetyltransferase activity was monitored continuously using a Tecan Infinite 200 microplate reader as described previously (Kim *et al.*, 2000 ▶). Briefly, the coenzyme A generated in a WcbI reaction was continuously measured using a coupled system with pyruvate dehydrogenase; the CoA-dependent oxidation of pyruvate is accompained by the reduction of NAD^+^ to NADH, which was measured spectrophotometrically at 340 nm. Reactions were performed in 50 µl and consisted of 10 m*M* HEPES pH 7, 0.2 m*M* NAD^+^, 0.2 m*M* thiamine pyrophosphate, 5 m*M* MgCl_2_, 1 m*M* dithiothreitol, 2.5 m*M* pyruvate and 0.03 U pyruvate dehydrogenase (Sigma–Aldrich), 1 m*M* GDP-mannose or 1 m*M* mannotetraose, 0.5 µ*M* recombinant WcbI and 0–500 µ*M* acetyl-CoA. Assays were performed in duplicate at 37°C, mixing all reagents without WcbI for 3 min to exhaust endogenous CoA; after this, WcbI was added and the reaction was followed for a further 5 min. The same assay was performed using 20 U chloramphenicol acetyltransferase (Sigma) and 1 m*M* chloramphenicol as a positive control.

## 3. Results   

### 3.1. Phasing   

The crystal structure of WcbI was determined by the multiple-wavelength anomalous dispersion method, using data collected at four wavelengths. Peak, inflection and high-energy remote data were collected to a limited resolution with short exposures and low transmission in order to minimize possible radiation damage and to collect multiple equivalents. As the WcbI crystals belonged to space group *P*1, a high-energy remote data set was collected over a rotation interval of 360° to increase the data redundancy. No radiation damage to the crystal was apparent after the first three data sets, so the exposure times were increased for the low-energy remote data set, which was collected over an interval of 360° to a high resolution of 1.56 Å. As the crystal proved to be stable to X-­rays and diffracted well, the reflections from corners of the detector were used in anomalous phasing, as the experiment geometry was the same for the different data sets. In spite of the low reported completeness of the peak, inflection and high-energy remote sets (Table 1 ▶), these data were over 90% complete to 2.7 Å resolution. The coordinates of 24 Br ions were used for phasing. These were located using 1000 attempts by *SHELXD* at 2.4 Å resolution with a correlation of 27.3% (20.5% at high resolution). The high resolution of the low-energy remote data set was crucial for subsequent density modification and successful building of the WcbI polyalanine model (605 residues out of 622) by *SHELXE*. For the correct hand, the correlation for the partial structure against the native data was 36.5%.^1^ The full WcbI atomic model was built by *ARP*/*wARP* using the polyalanine model produced by *SHELXE* and the low-energy remote data. This was found to contain bound CoA, carried through purification from the expression host, and will henceforth be termed the WcbI native structure. A second crystal was obtained co-crystallized with substrate acetyl-CoA, and high-resolution complex data were collected. The statistics of data collection and of model refinement and validation are presented in Table 1 ▶. 

### 3.2. Quality of the models   

The structure of the CoA complex of WcbI and the native structure were refined by *PHENIX* and *CCP*4, respectively, to resolutions of 1.38 and 1.56 Å, respectively. For each model the final round of refinement resulted in acceptable values of the *R* factor and *R*_free_. The WcbI crystals contained two independent molecules in the unit cell. Some residues were not modelled owing to poor electron density at the N-termini. The *G*-factors calculated for both models confirmed that the structures have normal stereochemical properties as given by *PROCHECK* (Laskowski *et al.*, 1993 ▶). The Ramachandran plots of the models (Chen *et al.*, 2010 ▶) revealed that at least 97% of the residues lie in the most favoured regions. The native structure contains 38 bromide ions. These were located using anomalous difference synthesis with peak Br data and phases from the refined model. Cofactor molecules were positioned in the active site using *F*_o_ − *F*_c_ OMIT maps and the occupancies of these molecules and the bromides were refined by *PHENIX*. In both subunits of both structures Ile244 is the sole Ramachandran plot outlier. This residue is well defined in the electron density and the *B* factors of its atoms are low. Many residues were modelled with alternative conformations of their side chains. For several regions in both structures the main chain was modelled in an alternative conformation. There are no WcbI residues with a *cis* conformation.

### 3.3. Overall structure   

The triclinic unit cell of the WcbI crystal contains two molecules (residues 2–310 for chain *A* and residues 4–310 for chain *B*). Comparison of the structure with extant structures using the protein structure comparison services *DALI* (http://ekhidna.biocenter.helsinki.fi/dali_server/; Holm & Rosenström, 2010 ▶) and *PDBeFold* (http://www.ebi.ac.uk/msd-srv/ssm; Krissinel & Henrick, 2004 ▶) revealed that WcbI largely adopts a novel fold. The fold consists of two sub­domains (Figs. 2 ▶ *a*, 2 ▶ *b* and 2 ▶ *c*). The N-terminal lobe (amino acids 2–102) forms a four-stranded parallel β-sheet with connectivity −1x, +2x, +1x (Richardson, 1981 ▶) flanked on one side by two α-­helices (α1–α2) and on the other by three α-helices (α3–α5). This arrangement has previously been observed in a number of proteins with different functions, for example *Neisseria meningitidis* glycerate kinase (New York SGX Center for Structural Genomics, unpublished work; PDB entry 1to6) and the zinc-transport protein ZnuA (Banerjee *et al.*, 2003 ▶; PDB entry 1pq4). Most of these proteins have a ligand/metal-binding site in the crevice formed at the C-termini of β1 and β3 of the β-­sheet. Cys14 of WcbI is located in this position. The C-­terminal subdomain (amino acids 103–311) is a novel arrangement of several α-helices that pack against the N-­terminal domain (Fig. 2 ▶ *a*). Analytical size-exclusion chromatography (Supporting Fig. S1) indicates that WcbI is likely to be monomeric in solution. The two molecules are related by an approximate twofold axis with a rotation angle of 175°; however, the buried surface area at the interface is only 140 Å^2^, which is less than 4% of the total accessible monomer surface area. Analysis of the structure with *PISA* did not indicate any likely multimerization interfaces preserved in the crystal.

### 3.4. Binding of acetyl-CoA and CoA to WcbI   

Analysis of early crystals of WcbI revealed that an unidentified ligand had been partially retained with the protein from expression in *E. coli* through the purification process (Supporting Fig. S2*a*). Mass spectrometry clearly identified CoA as the ligand co-purified with WcbI (data not shown). We therefore co-crystallized WcbI with acetyl-CoA. CoA was clearly defined in the electron-density map (Fig. 3 ▶ *a*) in both protomers, whilst acetyl-CoA was only observed in protomer *A*. However, following occupancy refinement, the occupancy of the acetyl moiety was too low for meaningful modelling. The molecule is located in a funnel-shaped cleft, surrounded by loops. The bound cofactor adopts a U-like overall conformation that is stabilized by several hydrogen bonds to WcbI, electrostatic interactions with charged residues, and hydrophobic contacts (Fig. 3 ▶ *b*, Supporting Fig. S2*b*). In both protomers, the β-mercaptoethylamine group of CoA forms a disulfide bond to the residue Cys14 (Figs. 3 ▶ *b* and 3 ▶ *c*), whilst the acetyl-CoA S atom is positioned near, but slightly away, from this and its carbonyl group is hydrogen-bonded to the S atom of Cys14 and His201 (data not shown). Interestingly, in the native structure the CoA terminal SH group points away from Cys14 toward Cys41 (Supporting Fig. S2*a*), perhaps owing to a more reducing environment of the crystallization conditions. Water molecules and Tyr97 form hydrogen bonds to the 4-phosphopantothenic acid portion of the acetyl-CoA (Fig. 3 ▶ *c*, Supporting Fig. S2*c*). The side chain of Arg294 is in electrostatic contact with the 3′-phosphate group; the amide N atoms of Ser69, Arg167 and Asn172 form hydrogen bonds to the 5′-phosphate group. Finally, water molecules form hydrogen bonds to the ADP moiety, which is also involved in a cation–π interaction with the guanidinium group of Arg167 (Fig. 3 ▶ *c*, Supporting Fig. S2*c*). The extensive interactions of CoA with both the N-terminal and C-­terminal domains is in contrast to the GNAT acetyltransferases that superficially resemble the N-terminal domain of WcbI, in which CoA is intimately bound to a single domain (Supporting Fig. S3).

### 3.5. Ligand- and cofactor-binding study by differential scanning fluorimetry   

**Figure 4:**
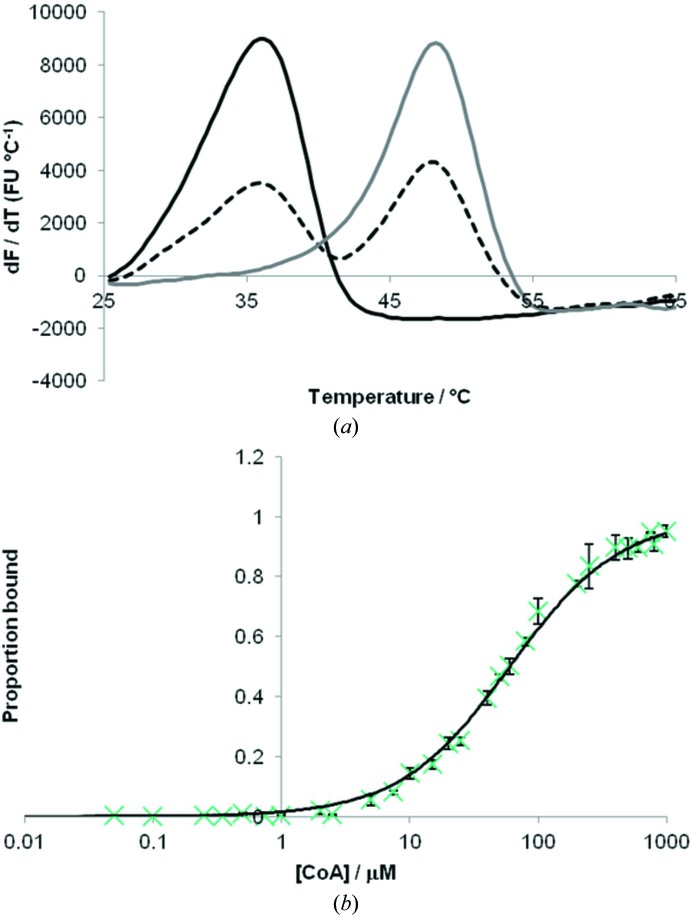
Biophysical characterization of WcbI binding to CoA. (*a*) Differential scanning fluorimetry thermal denaturation curves for WcbI show a biphasic response. With no CoA (black line) or a high saturation (1 m*M*) of CoA (grey line), a monophasic response is observed. At intermediate concentrations of CoA (60 µ*M*; dashed black line), a biphasic curve representing some bound and some unbound WcbI is observed. (*b*) WcbI shows dose-dependent binding to CoA, as expected for a single tight-binding molecule. All experiments were performed in triplicate and the data are representative of at least three experiments. The black line represents the fit of the data according to (1).

To verify that CoA is a genuine ligand for WcbI, we investigated its binding using differential scanning fluorimetry (DSF; Niesen *et al.*, 2007 ▶). Firstly, the protein was extensively dialysed in the presence of reducing agents to remove any disulfide-bonded CoA from the protein. The apoprotein was then incubated with a range of CoA concentrations and the thermal stability of the protein was probed. The presence of CoA showed a marked stabilizing effect, raising the apparent melting temperature by 10°C (Fig. 4 ▶ *a*), whilst a range of other common chemicals found in bacteria (after Niesen *et al.*, 2007 ▶) failed to give any significant change (data not shown). Fitting of the data (Fig. 4 ▶ *b*) indicated a dissociation constant *k*_d_ of 58 ± 2 µ*M*, which is well within the normal range of bacterial acetyl-CoA concentrations (Takamura & Nomura, 1988 ▶).

As *wcbI* is essential for the virulence of *B. pseudomallei* (Cuccui *et al.*, 2007 ▶), and the activity to acetylate the CPS has not been identified (Cuccui *et al.*, 2012 ▶), we hypothesized that WcbI might be this acetylase. To this end, using DSF, we investigated whether WcbI might bind to any potential substrates. We tried a range of simple sugars (d-mannose, d-­maltose, d-lactose, d-arabinose and d-lyxose), sugar polymers (cellubiose and polymannose) and nucleotides and sugar nucleotides (GDP, UDP and GDP-mannose). In each case there was no apparent alteration in the melting temperature (Table 2 ▶). It was apparent that it would not be possible to identify a potential substrate in this manner.

### 3.6. Acetyltransferase kinetic assays   

**Figure 5:**
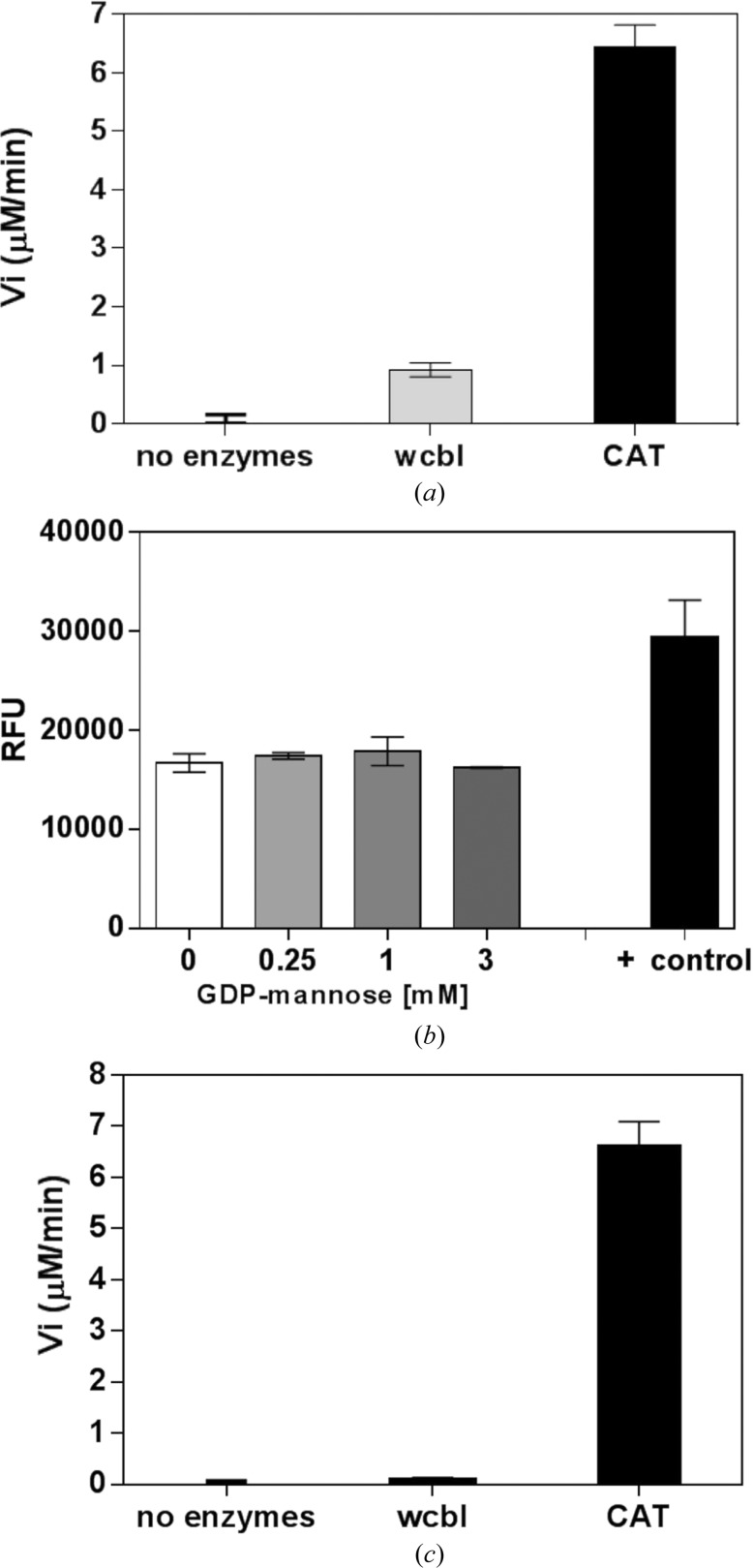
WcbI shows no detectable acetyltransferase activity. (*a*) Acetyltransferase activity was measured using the DTNB assay. Each column represents an average of three measurements. The concentrations of acetyl-CoA and GDP-mannose were held constant at 1 and 4 m*M*, respectively. The same assay performed with chloramphenicol acetyltransferase and 2 m*M* chloramphenicol served as a positive control. Reactions in which recombinant WcbI and CAT were omitted served as a negative control. (*b*) Acetyltransferase activity was measured using a fluorescence-based activity assay. Concentrations of GDP-mannose ranging from 0 to 3 m*M* were used, with the WcbI concentration held constant. The positive control supplied in the kit was used according to the manufacturer’s instructions. (*c*) Acetyltransferase activity was measured using a coupled assay with pyruvate dehydrogenase. Substrate concentrations were 0.5 m*M* for acetyl-CoA and 1 m*M* for GDP-mannose. The same assay was performed using CAT and 1 m*M* chloramphenicol as a positive control. A negative control was performed without either enzyme. The rates were calculated using the extinction coefficient for NADH. All columns represent the average ± standard deviation of two measurements.

To determine whether enzymatic assays could assist in determining a potential substrate for WcbI, we used several different assays to assess the capacity of the enzyme to acetyl­ate GDP-mannose or mannotetraose. These were selected as excellent analogues for the most likely substrates for WcbI: GDP-mannose is highly similar to GDP-6-deoxy-β-d-*manno*-heptopyranose, differing by one –CH_2_– group; whilst mannotetraose is a reasonable analogue of the polymerized sugar (having similar sugar units but a slightly different linkage). We attempted three chemically different assays for acetyltransferase activity. A DTNB-based assay (Riddles *et al.*, 1983 ▶), which measures the level of free thiols (and hence spent CoA), was performed. No significant acetylase activity was detected using GDP-mannose or mannotetraose as acceptor substrates. Whilst small changes in the product absorbance were observed, these were comparable to those seen in a bovine serum albumin negative control. Indeed, the background reaction rate increased with increasing acetyl-CoA or recombinant WcbI concentrations. WcbI carries over some CoA from purification, apparently forming a covalent thiol adduct with Cys14, as the crystal structures attest (Fig. 3 ▶ *a*). Similar adducts resulted in a catalytically inactive form in the case of glucosamine-6-phosphate *N*-acetyltransferase 1 (Dorfmueller *et al.*, 2012 ▶). This enzyme can be reactivated under reducing conditions with increasing amounts of reducing agents. To ascertain whether addition of reducing agents could rescue WcbI activity, we repeated this assay in the presence of DTT. Although there was a greater increase in the absorbance at 412 nm, this was also observed in controls and is likely to arise from the seven free cysteines of WcbI and the free CoA contaminant reacting with the DNTB molecule, suggesting that this assay is not suitable for this purpose. However, as expected, in the positive control performed using chloramphenicol acetyltransferase (CAT) the assay clearly shows a significant amount of CAT enzyme activity compared with WcbI (Fig. 5 ▶ *a*).

We therefore performed a second assay, specifically measuring free CoA using a fluorescent detection kit. Using a concentration range of GDP-mannose from 0 to 3 m*M* and constant WcbI and acetyl-CoA, there was no evidence for the transfer of the acetyl group to the substrate GDP-mannose compared with a control reaction (Fig. 5 ▶ *b*). A third, coupled assay was therefore performed. In this assay, spent CoA is turned over by pyruvate dehydrogenase, with concomitant reduction of NAD^+^ to NADH. This reaction can be followed spectrophotometrically at 340 nm in real time. The assay performed well in the presence of a test acetyltransferase (chloramphenicol acetyltransferase). However, using WcbI, no activity was observed using acetyl-CoA and either GDP-mannose or mannotetraose as substrates (Fig. 5 ▶ *c*). Furthermore, computational approaches based on analysis of protein sequence and structure, such as the 3*DLigandSite* (Wass *et al.*, 2010 ▶), *LIGSITE csc* (Huang & Schroeder, 2006 ▶), *ConCavity* (Capra *et al.*, 2009 ▶) or *FINDSITE* (Brylinski & Skolnick, 2008 ▶) servers, have been employed to predict potential further protein functional sites, including ligand-binding sites. All of these prediction servers confirmed the presence of a binding pocket for CoA, but did not identify any WcbI surface region suitable for interactions with sugars or sugar-nucleotides.

## 4. Discussion   

The *B. pseudomallei* capsular polysaccharide consists of a linear repeat of –3)-2-*O*-acetyl-6-deoxy-β-d-*manno*-heptopyranose-(1–. The genes required for the biosynthesis of this polysaccharide have been mapped in *B. pseudomallei* (Reckseidler *et al.*, 2001 ▶) and *B. mallei* (DeShazer *et al.*, 2001 ▶). Six genes (*wcbJ*, *wcbK*, *wcbL*, *gmhA*, *wcbM* and *wcbN*) form a unit that is proposed to synthesize the precursor sugar GDP-6-deoxy-β-d-*manno*-heptopyranose (dDHep) from sedoheptulose 7-phosphate, a pentose phosphate pathway intermediate (Cuccui *et al.*, 2012 ▶; Fig. 1 ▶). Nevertheless, the protein(s) that acetylates either GDP-6-deoxy-heptose or the partially polymerized capsule has not been identified. To date, WcbI has no predicted function (DeShazer *et al.*, 2001 ▶; Reckseidler *et al.*, 2001 ▶), but has been shown to be required for virulence (Cuccui *et al.*, 2007 ▶) and for the production of the capsule immunogen (Cuccui *et al.*, 2012 ▶).

The crystal structure of WcbI demonstrated that this protein largely adopts a novel fold (Fig. 2 ▶). The fold of the N-­terminal domain (amino acids 2–102) is common to several extant protein structures involved in signalling, ligand binding and nucleotide chemistry. The remaining 208 amino acids form an extended, largely α-helical structure, although a short portion also packs onto the N-terminal domain. It is striking that this fold generates a helical backbone that presents a loop-rich region at the interface between this domain and the N-terminal domain. The outcome is a large cleft between the two domains, with the promise of molecular flexibility to aid the ingress and egress of substrates, and perhaps to allow some remodelling of the binding site.

Surprisingly, our crystal structure of WcbI showed that the protein carries a ligand molecule through purification from *E. coli* (Supporting Fig. S2). Differential scanning fluorimetry demonstrates that this molecule is likely to be coenzyme A, as the presence of this molecule increases the apparent melting temperature of WcbI by approximately 10°C (Table 2 ▶, Fig. 4 ▶ *a*). The apparent *k*_d_ of 58 µ*M* is well within the physiological range for most bacteria, implying that this is a reasonable candidate for the substrate of the protein. Co-crystallization with 200 µ*M* acetyl-CoA led to almost complete occupancy of the ligand, with all CoA ligand atoms visible in the density. Although there is low occupancy of the acetyl group in the structure, the functional groups of acetyl-CoA form an extensive set of interactions with the protein, supporting the premise that this is a bona fide ligand of WcbI. It is therefore not unreasonable to hypothesize that WcbI could provide the missing acetyltransferase activity. A structural similarity search did not yield matches with other known acetyltransferases; the highest scoring *PDBeFold* hit (*Q* = 0.15; PDB entry 4h08, a putative hydrolase; Joint Center for Structural Genomics, unpublished work) and *DALI* hit (*Z* = 9.8; PDB entry 3hg7, a putative dehydrogenase; New York SGX Research Center for Structural Genomics, unpublished work) have three helices in a similar conformation but otherwise have no similar structure. Comparison with previously described GNAT acetyltransferases demonstrated that WcbI folds and binds CoA in a very different manner (Supporting Fig. S3).

To pursue this hypothesis, we performed kinetic and biophysical assays. In contrast to the results observed with CoA (Fig. 4 ▶ *a*), no thermal shifts were observed in the presence of potential substrates such as simple sugars (d-mannose, d-­maltose, d-lactose, d-arabinose and d-lyxose), sugar polymers (cellubiose and polymannose) or nucleotides or sugar nucleotides (GDP, UDP and GDP-mannose; Table 2 ▶). This was the case either in the presence or in the absence of acetyl-CoA, indicating that the binding of the first substrate does not create a site for a secondary substrate. These data do not unequivocally eliminate the possibility of a second substrate binding: there are cases in which a ligand may bind tightly without shifting the melting temperature of a protein, and larger *T*_m_ shifts are observed for more entropically driven compound binding (Vedadi *et al.*, 2010 ▶).

Given the quite clear binding of CoA to a protein required for capsule biosynthesis, the need for an acetyltransferase in the biosynthetic pathway and the suggestive conformation of the bound CoA, a detailed kinetic analysis was performed. We used three different biochemical assays to demonstrate the acetyltransferase activity of WcbI. None of these showed any detectable activity (Fig. 5 ▶). Assays were carried out under both oxidizing and reducing conditions, as previous studies on other acetyltransferases (Dorfmueller *et al.*, 2012 ▶) have shown that the formation of an oxidized adduct between Cys and CoA has an inhibitory effect on catalysis, which can be re­activated through reduction. No activity was apparent in either environment. This was somewhat surprising, as the structure shows CoA protruding into a funnel-shaped cavity (Supporting Fig. S4). Furthermore, the acetyl moiety is held in place through hydrogen bonds to Cys14 and His201, constraining its conformation and presenting the acetyl group in a suitable orientation for reaction.

Moreover, detailed analysis of the structure suggests that there is no available binding site for a reacting molecule. Analysis of the structure using four different servers suggested that the only likely binding site for a small molecule is that occupied by CoA. This is consistent with our observation that likely binding partners, and a range of common cellular chemicals, do not show any affinity for WcbI in DSF experiments. These observations, coupled with the lack of any distinguishable acetyltransferase activity, suggest that WcbI might form part of a more extensive enzymatic complex. We therefore propose a model in which WcbI functions together with a binding partner that presents the acceptor substrate. The nature of the CoA-binding pocket, as a loop-rich cradle in between two structured domains (Supporting Fig. S4), makes it more amenable to the likely subtle rearrangements that would be required to accommodate a presented ligand. In this model, we speculate that the complex between WcbI and its partner would transform the novel structure of WcbI into a catalytically active form upon association. Such modularity has been proposed as an ancestral trait providing robustness against mutation and chemical attack (Hartwell *et al.*, 1999 ▶), and promoting evolvability (useful for CPS formation and evolution in *B. pseudomallei*).

Our current investigations are aimed at identifying the identity of potential partners and understanding fully how this protein contributes to the virulence of *B. pseudomallei.*

## 5. Conclusions   

The *B. pseudomallei* protein WcbI, which is essential for the virulence of this organism, demonstrates an intriguing novel fold. This fold incorporates a previously observed subdomain into a larger folding unit, creating a loop-rich cradle-like structure. This cradle forms a strong binding site for coenzyme A, providing tight interactions for most of the cofactor. The two structures of WcbI presented here, obtained under different conditions, suggest that WcbI allows some flexibility in the CoA head group. Soaks with acetyl-CoA reveal that the CoA head, and likely the acetyl group, are held between cysteine and histidine side chains from opposite sides of the cradle in a conformation suggestive for reaction. However, extensive experiments on WcbI have failed to demonstrate either physical interaction with, or acetyltransferase activity on, available substrate analogues. We therefore propose that WcbI forms a module of a larger complex achieving the biosynthesis of the *B. pseudomallei* capsular polysaccharide.

## Supplementary Material

PDB reference: WcbI, bound to coenzyme A, 4bqn

PDB reference: native WcbI, 4bqo

Supporting information.. DOI: 10.1107/S205225251302695X/jt5001sup1.pdf

## Figures and Tables

**Table 1:** Data-collection and refinement statistics Values in parentheses are for the outer resolution shell.

	Br soak				
Data set	Low-energy remote	Peak	Inflection	High-energy remote	CoA complex
Beamline (Diamond Light Source)	I02	I02	I02	I02	I24
Space group	*P*1	*P*1	*P*1	*P*1	*P*1
Unit-cell parameters (Å, °)	*a* = 47.2, *b* = 67.9, *c* = 71.9, α = 62.9, β = 76.2, γ = 69.8	*a* = 47.2, *b* = 67.9, *c* = 71.9, α = 62.9, β = 76.2, γ = 69.8	*a* = 47.2, *b* = 67.9, *c* = 71.9, α = 62.9, β = 76.2, γ = 69.8	*a* = 47.2, *b* = 67.9, *c* = 71.9, α = 62.9, β = 76.2, γ = 69.8	*a* = 46.9, *b* = 68.0, *c* = 71.5, α = 63.0, β = 76.5, γ = 70.0
Rotation interval collected (°)	360	180	180	360	300
Wavelength (Å)	0.9795	0.9200	0.9202	0.9098	0.9778
Resolution range (Å)	63.7–1.56 (1.60–1.56)	63.7–1.96 (2.01–1.96)	63.7–1.86 (1.91–1.86)	63.7–2.01 (2.–2.01)	24–1.38 (1.42–1.38)
Completeness (%)	92.0 (72.6)	66.0 (12.5)	56.6 (2.1)	69.6 (16.8)	92.3 (59.4)
Redundancy	3.9 (3.7)	3.8 (3.6)	1.9 (1.5)	1.9 (1.9)	2.8 (2.5)
〈*I*/σ(*I*)〉	13.0 (2.2)	15.3 (2.0)	14.8 (2.1)	16.3 (2.4)	55.9 (5.7)
*R*_sym_ † (%)	4.8 (57.1)	4.4 (59.0)	3.2 (29.7)	5.1 (46.6)	2.4 (18.4)
*R*_cryst_ ‡ (%)	17.71				11.95
*R*_free_ (5% of total data) (%)	21.29				15.7
R.m.s.d., bond lengths§ (Å)	0.011 [0.019]				0.007 [0.020]
R.m.s.d., bond angles§ (°)	1.5 [2.0]				1.3 [2.0]
Wilson *B* factor¶ (Å^2^)	31.6				21.0
Average *B* factor (Å^2^)					
Protein	27.2				19.0
Solvent	37.9				35.0
Ligand CoA	36.8				18.9
Occupancy of ligand	0.5–0.6				0.80–0.88
Ramachandran plot analysis†† (%)					
Residues in most favoured regions	97.1				97.2
Residues in outlier regions	0.16				0.16
*MolProbity* score [percentile]	1.82 [64th]				1.87 [46th]

† *R*_sym_ = 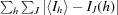 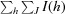, where *I*(*h*) is the intensity of reflection *h*, 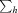 is the sum over all reflections and 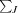 is the sum over *J* measurements of the reflection.

‡ *R*_cryst_ = 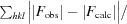 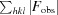.

§ Target values are given in square brackets.

¶ The Wilson *B* factor was estimated by *SFCHECK* (Vaguine *et al.*, 1999 ▶).

†† Ramachandran plot analysis was performed by *MolProbity* (Chen *et al.*, 2010 ▶).

**Table 2:** Thermodynamic analysis of the binding of sugars, sugar nucleotides and CoA to WcbI by DSF experiments

Ligands (1 m*M*)	*T*_m_ ± SD† (°C)
No ligand	37.4 ± 0.4
CoA	46.2 ± 0.2
Acetyl-CoA	45.6 ± 0.5
D-Mannose	37.0 ± 0.1
D-Maltose	37.1 ± 0.1
D-Lactose	37.5 ± 0.3
D-Arabinose	37.0 ± 0.1
D-Lyxose	37.1 ± 0.1
Cellubiose	37.7 ± 0.2
Polymannose	36.2 ± 0.1
GDP	37.0 ± 0.1
UDP	36.9 ± 0.05
GDP-mannose	38.2 ± 1.0

† The melting temperature *T*_m_ is expressed as the mean ± SD and was determined in triplicate. The concentration of WcbI was 0.11 mg ml^−1^.

## Footnotes

1 Correlations of over 30% at this stage generally indicate a correct solution.
