# Supplementary material for: Structural insights into WcbI, a novel polysaccharide-biosynthesis enzyme
Source: IUCrJ. 2013 Oct 18;1(Pt 1):28–38. doi: 10.1107/S205225251302695X (PMC4104973; doi:10.1107/S205225251302695X)
Supplement: Supplementary file 1 [file m-01-00028-sup1.pdf]

IUCrJ

Volume 1 (2014)

Supporting information for article:

Structural insights into WcbI, a novel polysaccharide-biosynthesis enzyme

Mirella Vivoli, Emily Ayres, Edward Beaumont, Michail N. Isupov and Nicholas J. Harmer

Vivoli, M., Ayres, E., Beaumont, E., Isupov, M. N. & Harmer, N. J. (2014). **IUCrJ**, **1**
doi:10.1107/S205225251302695X

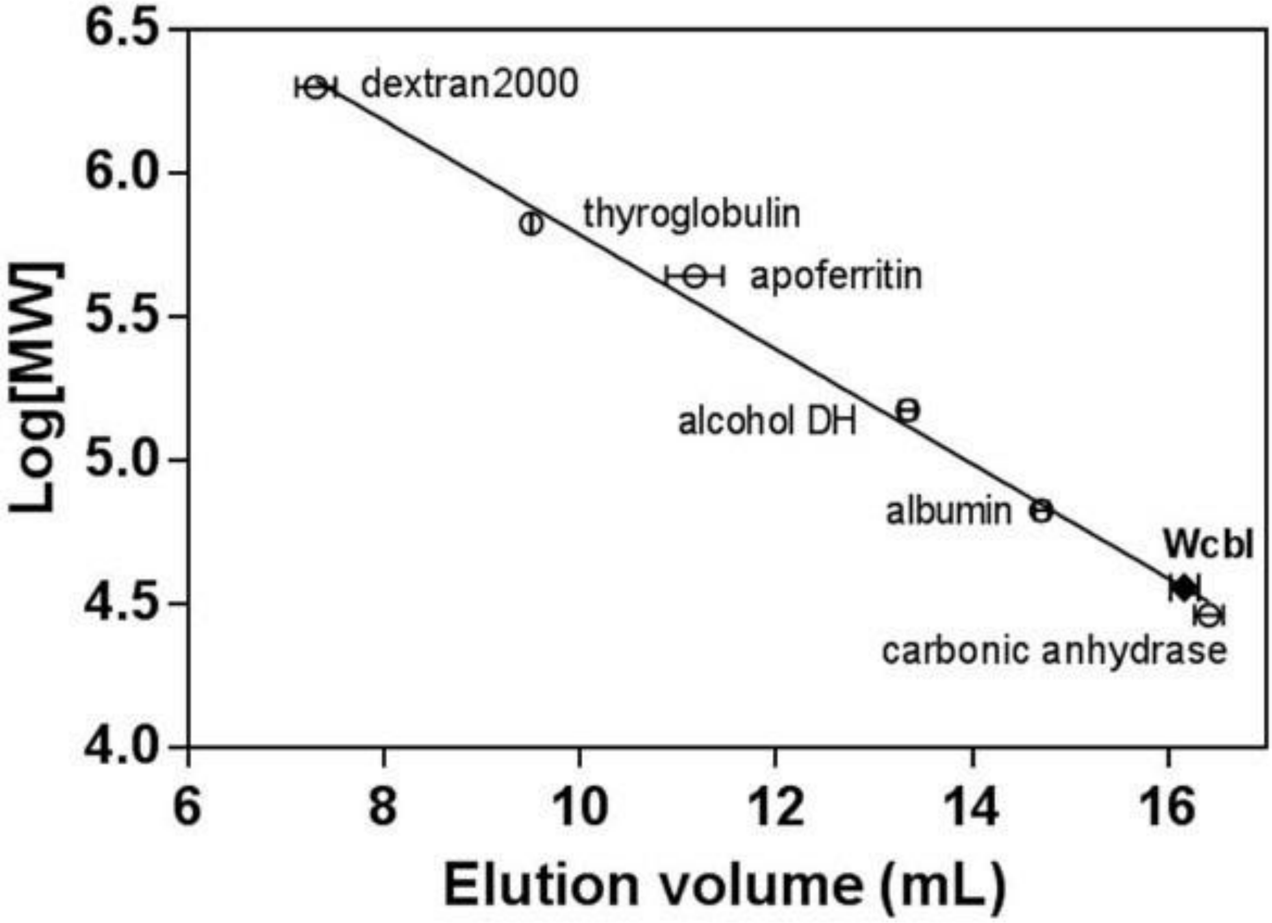

Figure S1 Analytical gel filtration chromatography of globular molecular weight standards: dextran 2000 (2,000 kDa), thyroglobulin (669 kDa), apoferritin (440 kDa), alcohol dehydrogenase (DH) (150 kDa), albumin (67 kDa), carbonic anhydrase (29 kDa) (empty circles). A Superdex 200 10/300 GL (24 mL bed volume; GE Healthcare) was run with 10 mM Hepes pH 7.0, 0.5 M NaCl at 4°C at a flow of 0.5 mL/min. Protein standards and WcbI were added at a concentration of 1.5 mg/mL. The logarithm of the molecular weight (LogMW) of the standard proteins obtained from three independent runs was plotted against the elution volume (mL). For clarity, the LogMW of WcbI obtained from three independent runs under the same running conditions is shown as black rhombus. A relative molecular weight of about 36 kDa was obtained from the standard curve, indicating a monomeric state of the protein in solution.

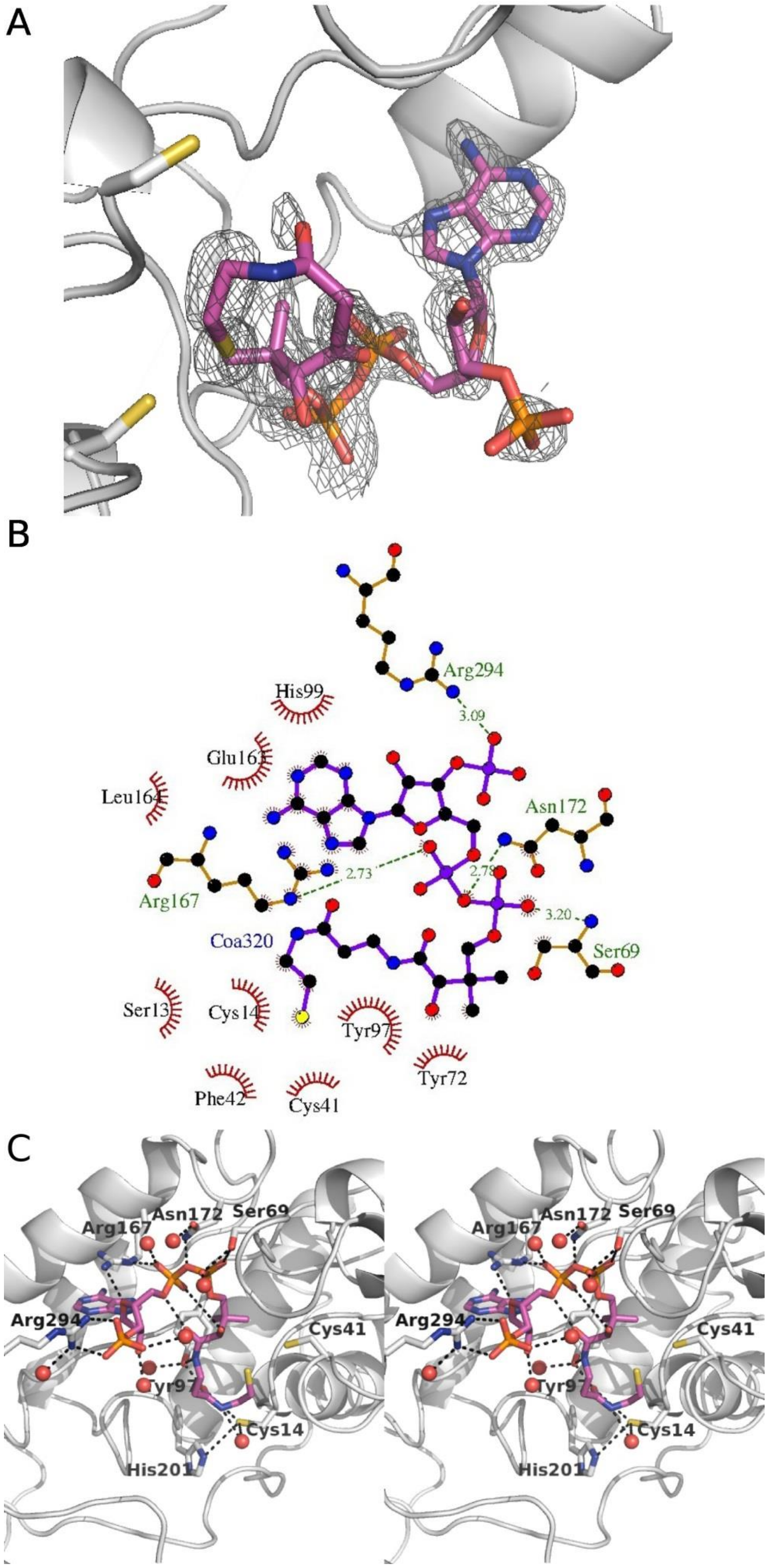
A
B
Arg294
3.09
His99
Glu16
Leu164
Asn172
2.73
2.78
Arg167
3.20
Coa320
Ser69
Ser13
Cys14
Tyr97
Tyr72
Phe42
Cys41
C
Arg167
Asn172
Ser69
Arg294
Cys41
Tyr97
Cys14
His201

Figure S2 **A.** Electron density map showing the presence of Coenzyme A (CoA) molecule in the native structure of WcbI, which was partially retained during the purification process. WcbI is shown as grey cartoon, CoA, Cys^{14} and Cys^{41} as sticks. For clarity, water molecules are not shown. An F_o-F_c omit map is shown contoured to 3σ. **B.** Schematic drawing of the WcbI-CoA interactions. **C.** Key interactions of WcbI-CoA interactions. WcbI is shown as cyan cartoon, with interacting side chains as purple sticks. CoA shown as sticks. Hydrogen bond distances are shown. Colours: nitrogen, blue; oxygen, red, phosphorus, orange, sulphur, yellow; CoA carbon, purple; WcbI carbon, grey. Images A and C were prepared with the Pymol Molecular Graphics System, and image B with $LigPlot^+$ (Laskowski & Swindells, 2011).

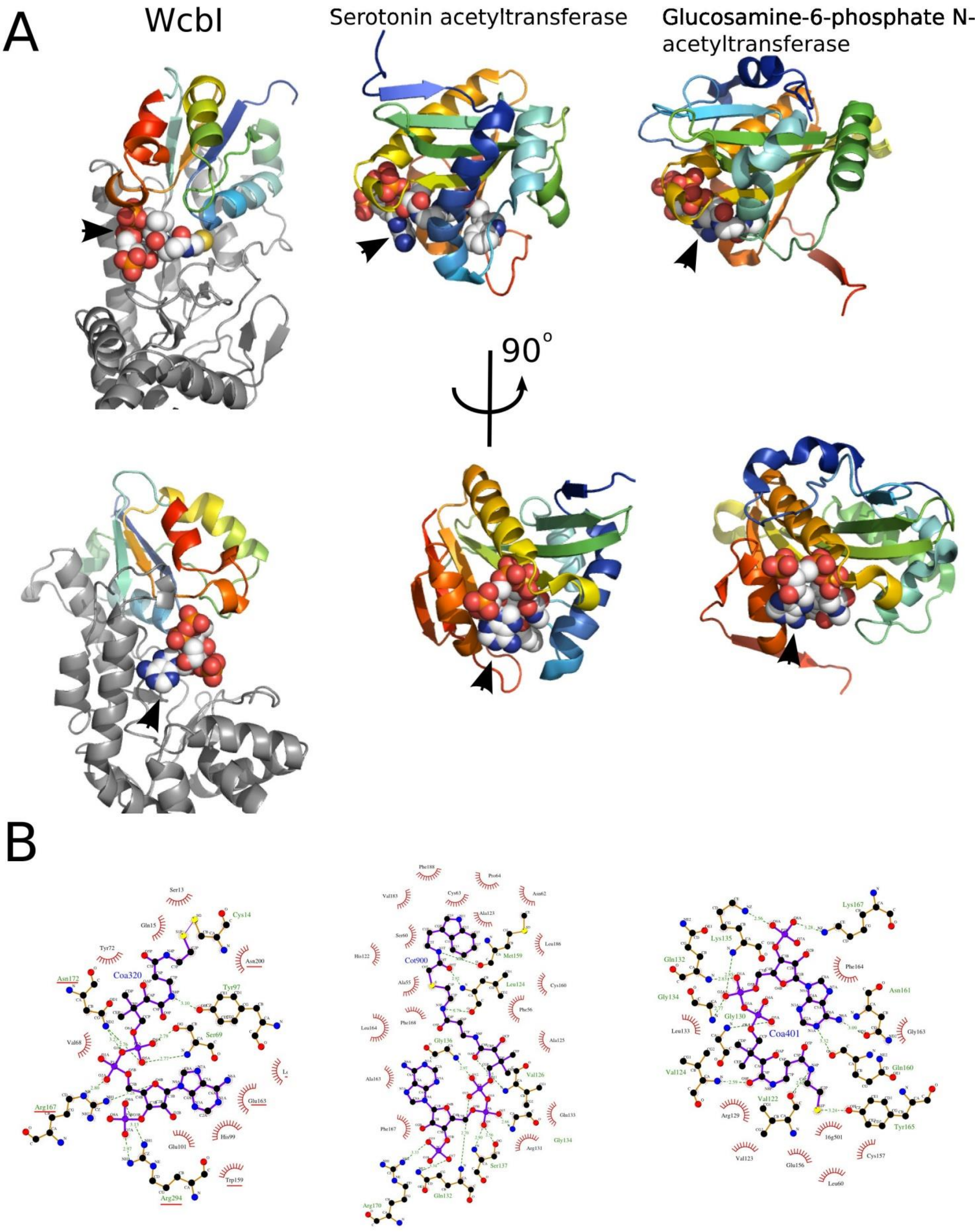

Figure S3 WcbI forms a novel fold, unrelated to previously observed acetyltransferases. The precedented N-terminal domain of WcbI shows a superficial resemblance to the GNAT family of acetyltransferases. Two representative GNAT acetyltransferases with solved structures in complex with CoA were superimposed

onto the structure of WcbI. Image A shows WcbI (left), human serotonin acetyltransferase (middle; PDB ID 1LOC; Schiebner *et al.*, 2002) and human glucosamine 6-phosphate *N*-acetyltransferase (right; PDB ID 2O28; H. Wu, J. Min, H. Zeng, P. Loppnau, J. Weigelt, M. Sundström, C. H. Arrowsmith, A. M. Edwards, A. Bochkarev, A. & A. N. Plotnikov, unpublished work) in two orientations, rotated by 90° around the y-axis. Protein is shown in cartoon form, coloured in a rainbow with the N-terminus blue, and the C-terminus red. For WcbI, the N-terminal domain is coloured as rainbow, and the remainder of the protein grey. CoA (or analogue) is shown as a full sphere model, with atoms coloured as follows: carbon, white; nitrogen, blue; oxygen, red; phosphorus, orange; sulfur, yellow. The adenine ring is indicated by a black arrowhead in each case. Notably, the N-terminal domain of WcbI forms a four-stranded, parallel β-sheet, whilst the GNAT acetyltransferases form a mixed parallel/antiparallel sheet with a ↑↓↑↑↓↑ pattern. Furthermore, the GNAT acetyltransferases interact extensively with all faces of CoA, whilst CoA binding by WcbI is shared between the N-terminal domain and the novel C-terminal domain (especially, for example, the 3' phosphate of CoA). **B**: Schematic drawing of the CoA interactions of each protein. In WcbI, interacting residues from the novel domain are highlighted by red underlining. Image A was prepared with the Pymol Molecular Graphics System, and image B with LigPlot+ (Laskowski & Swindells, 2011).

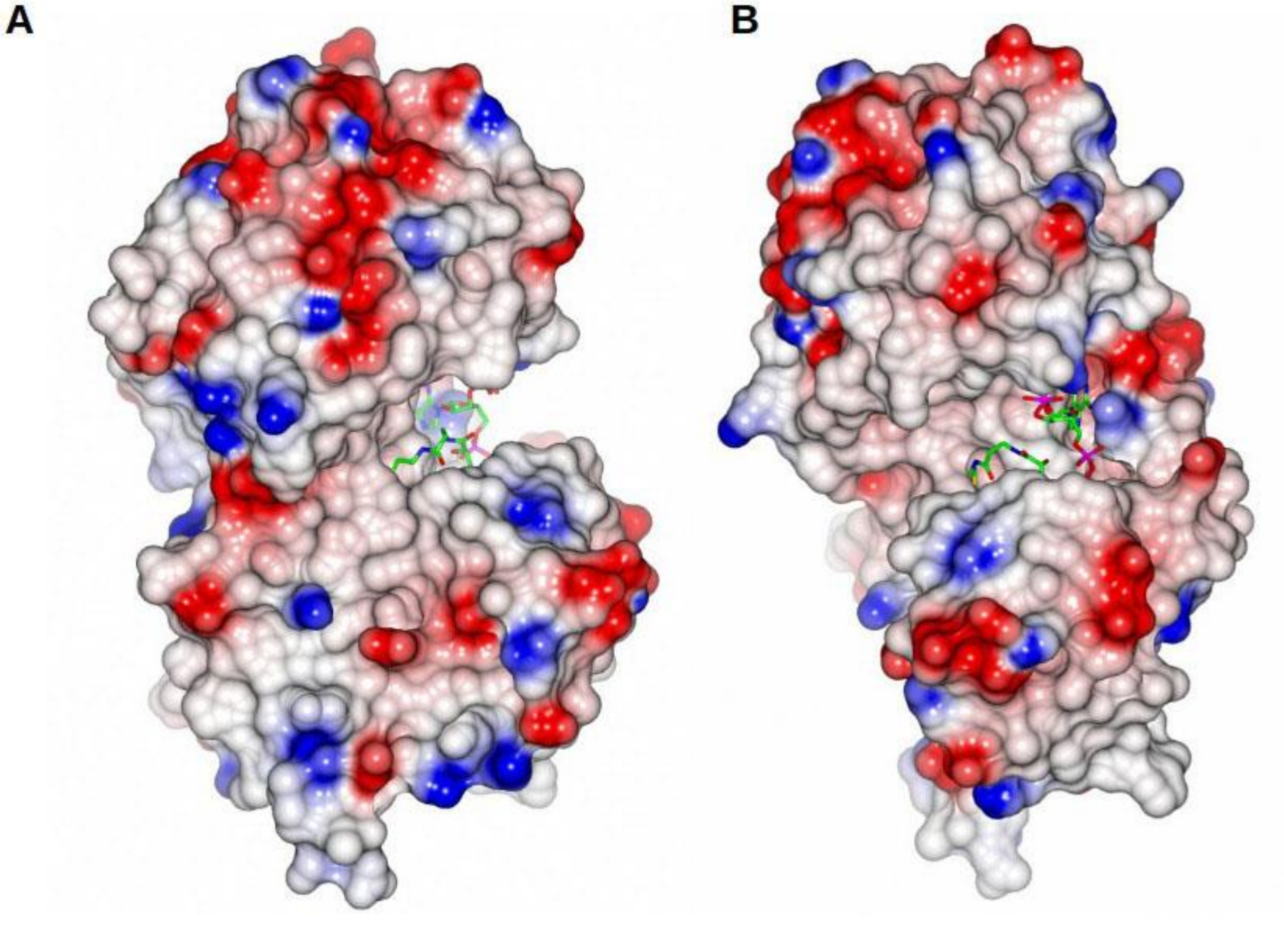

Figure S4 Molecular surface of WcbI structure with bound CoA. The surfaces are colored by electrostatic potential: basic, blue; acidic, red; and uncharged polar, white. CoA is represented as sticks (carbon atoms in

green). Surface was drawn and colored using ccp4mg (McNicholas *et al*., 2011). Notice the cradle between the two domains that allow the binding of CoA and the lack of any potential WcbI surface region favourable for interactions with sugars or sugar-nucleotides.

References

Laskowski, R. A. & Swindells, M. B. (2011). *J. Chem. Inf. Model*. **51**, 2778-2786.

McNicholas, S., Potterton, E., Wilson, K. S. & Noble, M. E. M. (2011). *Acta Cryst*. D**67**,386-394.

Scheibner, K. A., de Angelis, J., Burley, S. K. & Cole, P. A. (2002) *J. Biol. Chem.* **277**, 18118-18126.
